# A Systematic Review of Psychosocial Barriers and Facilitators to Smoking Cessation in People Living With Schizophrenia

**DOI:** 10.3389/fpsyt.2018.00565

**Published:** 2018-11-06

**Authors:** Alistair Lum, Eliza Skelton, Olivia Wynne, Billie Bonevski

**Affiliations:** School of Medicine and Public Health, University of Newcastle, Newcastle, NSW, Australia

**Keywords:** systematic review, barriers, facilitators, smoking cessation, schizophrenia, psychosocial

## Abstract

**Background:** People living with schizophrenia are less likely to quit smoking compared with the general population and people living with other psychiatric disorders. Understanding the schizophrenia-specific psychosocial barriers and facilitators to smoking cessation is important for designing effective smoking cessation interventions. We aimed to systematically review research examining psychosocial barriers and facilitators to smoking cessation in people living with schizophrenia.

**Methods:** We followed the PRISMA statement to conduct a systematic literature review examining psychosocial barriers and facilitators to smoking cessation in people living with schizophrenia. We searched EMBASE, Medline, PsycINFO, and CINAHL databases from inception to 14 June 2018 to identify relevant articles. We included peer-reviewed original research articles that examined psychosocial barriers and facilitators to smoking cessation, as well as factors associated with maintenance of smoking habits in people living with schizophrenia spectrum disorders. Qualitative, quantitative, or mixed-methods study designs were included. Three authors screened titles, abstracts, and full-texts using the eligibility criteria. We conducted a narrative synthesis of the data to account for the heterogeneity of study designs. We analyzed qualitative and quantitative studies separately.

**Results:** We identified 685 studies from our systematic search and screened the full-text of 134 articles. The final set of 23 articles included 20 quantitative studies and 3 qualitative studies. The most commonly cited barrier to smoking cessation in people living with schizophrenia was cravings and addiction, followed by a perceived increased risk of negative affect associated with quitting smoking. People living with schizophrenia reported smoking to manage stress and to maintain social relationships. People living with schizophrenia were found to be less likely to receive cessation support from health professionals than smokers without schizophrenia. Health concerns were the most commonly mentioned facilitator to quit smoking.

**Conclusions:** People living with schizophrenia experience a wide range of barriers to smoking cessation. The influence of these barriers on smoking cessation likelihood may be greater among people living with schizophrenia than people without psychiatric disorders. Health professionals play an important role in smoking cessation for people living with schizophrenia and should consider barriers and facilitators identified in this review to support quitting in this vulnerable population.

## Introduction

Approximately 65% of people living with schizophrenia smoke cigarettes (1, 2). People living with schizophrenia are significantly less likely to quit smoking compared with the general population and those living with other psychiatric disorders, such as bipolar disorder and depression (1). Given the elevated prevalence of tobacco smoking and low cessation rates, people living with schizophrenia are at higher risk of developing smoking-related malignancies, cardiovascular disease, and respiratory disease and are more likely to experience premature mortality than the general population (3). Improving smoking cessation rates in people living with schizophrenia will be integral to improving the health of this vulnerable population. Clinical Practice Guidelines for the treatment of tobacco smoking suggest clinicians provide a dual approach consisting of both pharmacological (e.g., bupropion, varenicline, and nicotine replacement therapy [NRT]) and psychosocial (e.g., cognitive behavioral therapy [CBT], motivational interviewing [MI], information and education interventions, and social support) strategies for people living with schizophrenia (4–6).

Research examining the pharmacological treatments for tobacco smoking among people living with schizophrenia exceeds the quantity of corresponding psychosocial research, potentially limiting the depth and breadth of smoking cessation advice that clinicians can provide. Pharmacological interventions appear to be effective with few safety concerns. A meta-analysis of five trials comparing bupropion or bupropion and NRT with placebos or placebos with NRT found that participants in the bupropion group were almost three times more likely to abstain from smoking at 6 month follow-up compared with those in the placebo group (7). Two trials that compared varenicline with placebo found almost five times greater smoking cessation rates in the varenicline group at end of treatment (7). Pharmacological smoking cessation interventions appear to be appropriate for people living with schizophrenia, however the percentage of successful quitters is reportedly small, between 12 and 19% (7).

In line with clinical guidelines, many randomized clinical trials of pharmacological interventions were delivered alongside psychosocial strategies for treatment of tobacco smoking (7). Yet, few trials assessed the benefits associated with psychosocial strategies in these combined interventions. The limited number of combined trials have shown limited long-term effect. One such trial compared a smoking cessation program founded on CBT and MI principles plus NRT with usual care plus NRT, and found higher smoking reduction rates in the intervention group at 3 months, but no differences in smoking abstinence or reduction rates beyond 6 months (8). Contingency reinforcement using money with or without NRT or bupropion have been associated with higher smoking abstinence compared with a minimal intervention group (9) and smoking reduction rates compared with a pharmacotherapy only group (10), however long-term effects were not reported.

Very few randomized controlled trials of psychosocial interventions for smoking cessation or reduction in people living with schizophrenia have been conducted (11–14). Psychosocial programs for smoking cessation in people living with schizophrenia have used a variety of approaches, including psychoeducation, MI, CBT, social skills training, relapse prevention, monetary contingent reinforcement strategies, or a combination of these approaches (15). One study compared a high-intensity program incorporating MI, social skills training, NRT education, and relapse prevention with a moderate-intensity program focused on medication compliance and NRT education (13). Smoking cessation rates did not differ between groups, with 21% abstinent at 12-weeks after the target quit date, 17% at 6 month follow-up, and 14% at 12 month follow-up. A comparison of the American Lung Association (ALA) smoking cessation program with a schizophrenia-targeted program comprised of MI, psychoeducation, and relapse prevention strategies found significantly higher abstinence rates in the ALA group at 6-month follow-up (11). The 6 month smoking cessation rates of the psychosocial interventions described above range between 11 and 18% (11, 13), which are comparable to rates achieved in pharmacotherapy trials, which range between 12 and 19% (16–18).

Research examining the neurobiological factors associated with smoking maintenance among people living with schizophrenia is substantial and continues to grow (19). This research is helping inform advances in pharmacological treatment options (19). In contrast, the theory underlying psychosocial interventions for people living with schizophrenia has not been well-defined in the literature, which may limit the effectiveness of psychosocial interventions for smoking cessation for people living with schizophrenia. Ziedonis and George (20) reviewed the literature on smoking cessation and schizophrenia prior to the development and evaluation of a psychosocial intervention promoting smoking cessation in people living with schizophrenia. However, their review of neurobiological and clinical issues failed to address psychosocial factors associated with smoking cessation consistent with their psychosocial intervention. Steinberg and Williams (21) examined the necessary modifications to treatment components of smoking cessation programs to better match the needs of people living with schizophrenia. They found that people living with schizophrenia may require more intervention sessions or sessions over a longer duration, content delivery adaptations to account for neurocognitive deficits common in people living with schizophrenia and social skills training (21). While these findings are important, this examination of intervention factors does not account for barriers of smoking cessation in people living with schizophrenia identified in the non-intervention literature.

Current reviews on barriers to smoking cessation in people living with schizophrenia have included people with other mental illnesses, such as bipolar disorder or severe depression. Common individual barriers to smoking cessation identified in these reviews include the desire to manage stress and avoid withdrawal symptoms, and the belief that smoking provides a sense of identity. Many people living with mental illness are not given cessation support from health care providers (22, 23). Smoking tobacco is often socially accepted among people living with mental illness (23, 24). Current evidence on facilitators of smoking cessation among people with schizophrenia, including perceived health, and financial benefits and social support for quitting, are also commonly experienced by people with other mental illnesses and the general population (23, 25).

Schizophrenia-specific barriers and facilitators to smoking cessation are less well-understood than those affecting people with all mental illness. One commonly mentioned reason for smoking among people with schizophrenia is the desire to manage negative symptoms (23, 24, 26). Managing negative symptoms, such as negative affect, anhedonia, and loss of motivation, can improve social and vocational functioning among people living with schizophrenia (27). While this self-medication hypothesis is recognized as a schizophrenia specific barrier to smoking cessation, continued efforts to determine other barriers and facilitators are required to ensure comprehensive support is available (28).

A stronger understanding of schizophrenia-specific barriers and facilitators to smoking cessation, such as reduction of negative symptoms, is required to help clinicians to provide optimal treatment options and intervention developers to better tailor their programs to the unique needs of people living with schizophrenia (28–31). Currently, psychosocial interventions have been developed without consideration of the full range of psychosocial barriers and facilitators. Thus, we cannot be confident that current interventions adequately address the unique psychosocial factors contributing to smoking cessation in people living with schizophrenia. Therefore, we aimed to systematically review research examining the psychosocial barriers and facilitators to smoking cessation in people living with schizophrenia. We asked two research questions:

1. What psychosocial barriers and facilitators affect smoking cessation in people living with schizophrenia?
2. Do people living with schizophrenia experience more psychosocial barriers that affect smoking cessation than people without mental illness?

## Methods

### Design

We systematically reviewed original research examining psychosocial barriers and facilitators to smoking cessation in people living with schizophrenia. The PRISMA statement guided the conduct of the review (32). We used the Covidence software in screening articles (33). We registered the review with Prospero (CRD42018103332).

### Search strategy

We searched EMBASE, Medline, PsycINFO, and CINAHL databases using keywords from inception to 14 June 2018 to identify relevant articles. Our search terms were [smoking OR tobacco OR cigarette OR nicotine OR e-cig] AND [schizophrenia OR psychosis OR schizoaffective OR schizophreniform OR delusional disorder OR psychotic OR psychoses] AND [factor$ OR determinant$ OR variable$ OR covariable$ OR predictor$ OR barrier$ OR facilitator$] AND [smoking cessation OR quitting smoking OR abstinence OR withdrawal OR quit$]. $ indicated truncation.

### Eligibility

We included peer-reviewed original research articles that examined psychosocial barriers and facilitators to smoking cessation, as well as factors associated with maintenance of smoking habits, in people living with schizophrenia spectrum disorders. Articles that included mixed diagnosis samples composed of 50% or more of participants with schizophrenia were included. We included participants living with schizophrenia as well as healthcare providers working with people living with schizophrenia in 50% or more of their cases. We included articles that reported outcomes relating to people who identify as smokers without a requirement that the study define the smoking status of participants. This inclusive approach was designed to increase the number of studies included in our review. We included qualitative, quantitative, and mixed-methods study designs. Cross-sectional and longitudinal quantitative study designs with or without a comparison group were eligible for this review. We chose to include studies with baseline data from intervention trials to increase the amount and quality of relevant data, yet the results must be interpreted with caution due to the potential bias associated with recruitment into an intervention trial. Articles published in gray literature or in languages other than English were excluded.

### Study selection

Two authors (AL and ES) simultaneously screened titles and abstracts using the eligibility criteria. All articles not excluded were screened using the full text by one author (AL), with two-thirds of articles screened by a second author (ES or OW). These three authors discussed any conflicts in screening.

### Data extraction

One author (AL) extracted data from all included articles using a standardized pre-piloted data extraction form. Data extracted included age, sex, study design, country of study, sample size, control group characteristics, diagnostic characteristics (e.g., recruitment site, percentage of sample which was diagnosed with schizophrenia, and criteria to assess diagnosis), smoking characteristics (i.e., number of cigarettes smoked daily, age smoking commenced, tool to assess smoking status, and nicotine dependence), barrier and facilitators characteristics (i.e., maintenance factors relating to smoking or barriers or facilitators to smoking cessation, assessment tool to measure factors, and relationship to outcome measure) and outcome measure (i.e., type of smoking outcome and assessment tool of outcome).

### Quality appraisal

One author (AL) critically appraised the risk of bias in all articles using the QualSyst tool (34). The QualSyst tool was developed as a tool designed to measure the risk of bias in a range of studies, including randomized trials and quantitative, qualitative, and mixed methods studies. Two checklists with manuals to guide scoring are available; one for quantitative studies with 14 items and one for qualitative studies with 10 items. We report the percentage of checklist items met for all studies to improve interpretability, with higher percentages indicating lower risk of bias.

### Data analysis

We conducted a narrative synthesis of the data to account for the heterogeneity of study designs. We synthesized data on barriers and facilitators to quitting and motivators to smoke. We analyzed qualitative and quantitative studies separately. Due to the lack of theory underlying psychosocial barriers and facilitators to smoking cessation in people living with schizophrenia, we took an inductive approach to data synthesis.

## Results

### Search results

**Figure 1:**
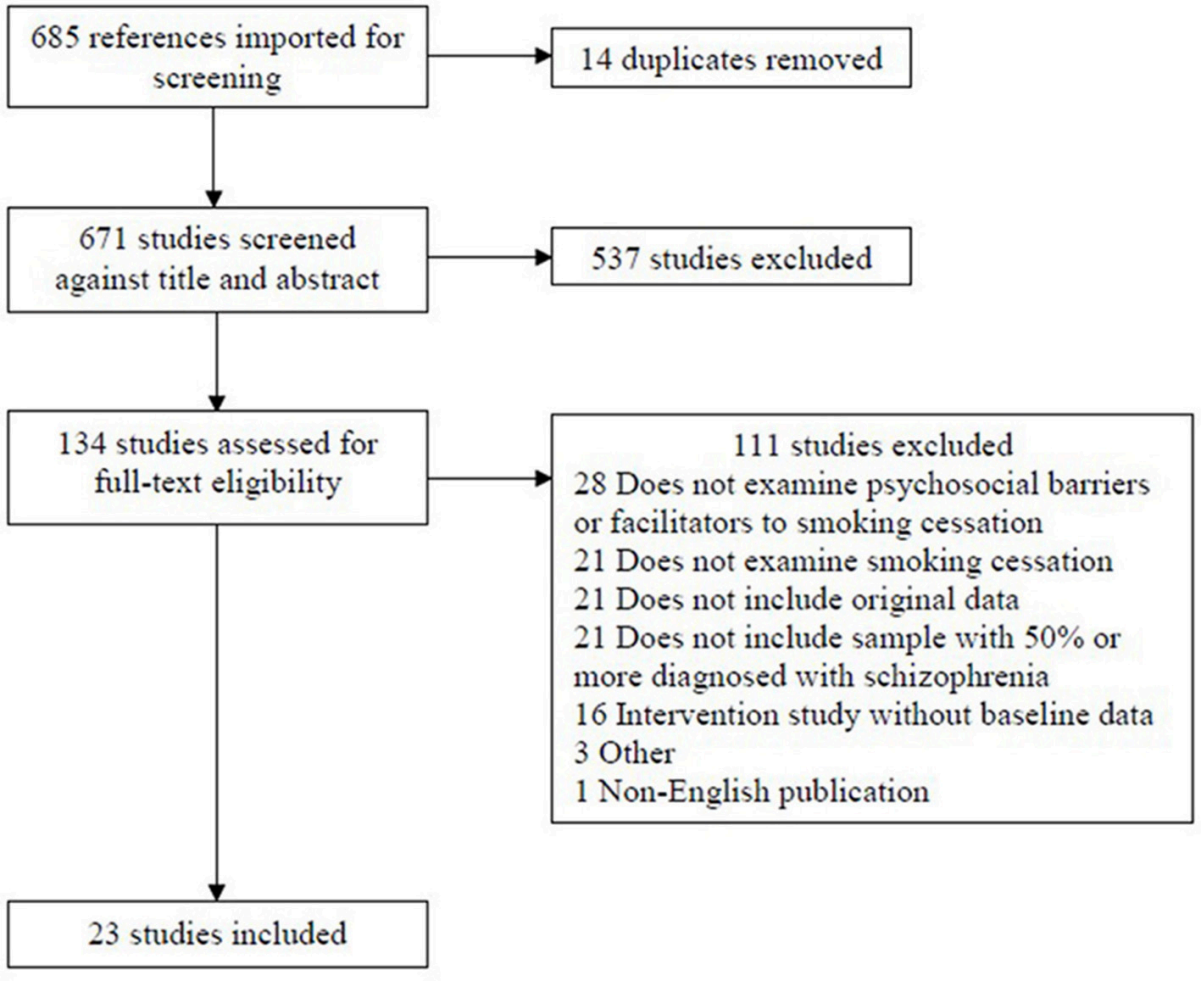
Flow diagram of screening process and outcomes.

We identified 685 studies from our systematic search, of which 14 were duplicates. We removed 537 of the 671 articles based on titles and abstracts that indicated the article was not relevant to our aims. We screened the full-text of the remaining 134 articles, and removed a further 111. The main reasons for excluding articles at the full-text screening stage were that the article did not examine psychosocial barriers or facilitators to smoking cessation, did not examine smoking cessation, did not present original data, or did not include a sample comprised of at least 50% of participants with schizophrenia (see Figure 1). The final set of articles included 23 articles, of which 20 had quantitative designs and 3 had qualitative designs.

### Study characteristics

Of the 3,557 participants in included studies, 3,257 had a diagnosis of schizophrenia (91.6%). All articles were published between 1996 and 2017. Eleven (48%) articles were published in the United States, five (22%) in Australia, three (13%) in Canada and one each in Turkey, Greece, Israel, the United Kingdom, and Scotland (4%). Of the 20 quantitative studies, 12 (60%) had cross-sectional designs, 6 (30%) examined baseline data from intervention trials, 1 (5%) was a non-randomized within-group trials, and 1 (5%) was a non-randomized controlled trial.

The quantitative articles met between 50 and 88% (median = 80%) of the QualSyst risk of bias criteria, while the qualitative articles met between 45 and 85% of the criteria (median = 85%). Seventy percent of quantitative (14/20) and 67% of qualitative (2/3) studies met 75% or more of the QualSyst criteria. These outcomes indicate that the combined data has a low risk of bias and the findings can be considered as reliable indicators of barriers and facilitators to smoking cessation in people living with schizophrenia. The majority of quantitative studies met the QualSyst criteria of reporting study objectives, sufficiently disclosing participant characteristics, recruiting an appropriate sample size, describing methods of data analysis, reporting an estimate of variance, reporting results in sufficient details, and drawing conclusions supported by the results. Outcome variables and method of participant selection were inconsistently reported with sufficient detail and study design was often not explicitly reported. The context of the study, sampling strategy, data analysis techniques, and coding biases were only partially described in at least two of the three qualitative studies. Study and participant characteristics, QualSyst scores, and a summary of key study outcomes are presented in Table 1 for quantitative studies (see sections Cravings and Addiction, To Reduce Negative Affect, Social Facilitation, Stress Management, Concern for Health Risks, Physician Advice to Quit Smoking, Systemic Barriers, Social Pressure to Quit, and Additional Barriers and Facilitators to Smoking Cessation) and Table 2 for qualitative studies (see section Qualitative Findings).

**Table 1:** Quantitative study characteristics and key findings.

**First author; year of publication; country**	**Study aims**	**Total sample size; N of relevant groups; sample size and group composition**	**Age in years (SD)**	**N male (%)**	**Assessment of barrier**	**Key findings**	**QualSyst score (%)**
Baker; 2007; Australia	To describe demographic and clinical characteristics, smoking behaviors, stage of change, and reasons for smoking and quitting in community-residing smokers with a psychotic disorder; and to compare smoking behaviors in this sample with data reported for other samples.	298; 3 groups; G1 = 298 (56.7% schizophrenia/ schizoaffective disorder, 9.1% bipolar disorder with mania, 6.4% severe depression with psychosis, 27.9 other psychosis), G2 = 387 (General population from Pederson et al. study), G3 = 1,215 (volunteer sample of smokers in smoking cessation study from Curry et al. study)	G1 = 37.24 (11.09), G2/G3 unknown	G1 = 156 (52.3%), G2/G3 unknown	Reasons for Smoking Questionnaire; Reasons for Quitting scale; Readiness and Motivation to Quit Smoking Questionnaire	Reasons for smoking: Stress reduction: G1 > G2; Stimulation: G1 > G2; Addiction G1 > G2; Reasons for quitting: Desire for self-control G1 > G3; Desire for immediate reinforcement: G1 > G3; Social influence G1 > G3; Health concerns G1 = G3.	83
Briskman; 2012; Israel	To compare preventative intervention and treatment rates for comorbidities in hospitalized patients with psychiatric illness with patients without psychiatric illness.	192; 2 groups; G1 = 93 (88% schizophrenia/ schizoaffective disorder, 8% bipolar disorder, 4% other), G2 = 99 (100% hospitalized patients without psychiatric illness)	G1 = 53.3 (15.1), G2 = 55.2 (14.3)	G1 = 47 (51%), G2 = 55 (56%)	Reported receipt of instruction to quit smoking	Received physician instruction to quit: G1 < G2.	58
Brown; 2015; USA	To examine effectiveness of psychiatrists implementing the 5 A's on smoking rates among clinic patients diagnosed with serious mental illness.	49; 1 group; G1 = 49 (100% mental health clinicians)	NA	NA	Clinicians' attitudes and beliefs regarding smoking cessation treatments and the 5 A's	Main perceived barriers to carrying out 5A's: Lack of interest among patients about smoking and/or smoking cessation; Too many demands on staff already to begin a new practice; Too time demanding to carry out 5 A's; Staff skepticisms of 5 A's.	79
Brunette; 2017; USA	To examine age differences on smoking habits and examine age, gender, attitudes and beliefs, social norms, and perceived behavioral control in smokers with schizophrenia in relation to intention to quit and use of cessation treatment.	184; 1 group; G1 = 184 (100% schizophrenia)	42.96 (12.7)	132 (71.4%)	Attitudes toward smoking scale; Self-developed theory of planned behavior questionnaire; Stigma of smoking questionnaire; Stages of Change questionnaire	Perceived adverse effects of smoking were greater than benefits and pleasures of smoking. Positive attitude to NRT or smoking cessation medication associated with higher intention to quit.	79
Coletti; 2015; USA	To assess smoking-related knowledge and the effects of health messages on smoking knowledge and behavior.	148; 2 groups; G1 = 69 (100% schizophrenia), G2 = 79 (100% healthy controls)	G1 = 22.67 (5.2), G2 = 27.97 (5.6)	G1 = 55 (79.7%), G2 = 30 (38.0%)	Self-developed smoking knowledge questionnaire; Valence and Arousal items of Self-Assessment Manikin	Health concerns: G1 < G2 Smoking warning's effectiveness: G1 = G2.	83
Duffy; 2012; USA	To estimate the prevalence of tobacco use and receipt of cessation services among Veterans Affair patients with mental illness, and determine the clinical, treatment, and demographic factors associated with receipt of cessation services.	224,193; 2 groups; G1 = 1,430 (100% smokers with schizophrenia), G2 = 27,652 (100% smokers without mental disorder)	Total sample = 3.4% < 45 years, 37.4% 45 to 64, 59.2% ≥65	NA	Reported receipt of advice to quit smoking by physician, provision of medication, and discussion of quitting methods	Received physician advice to quit: G1 < G2 Recommendations for smoking cessation medications: G1 = G2; Physician discussed quitting methods: G1 = G2.	71
Filia; 2011; Australia	To examine CHD-related behavioral risk factors in smokers with schizophrenia, their reasons for engaging in risky behaviors, and level of motivation and confidence to change.	43; 1 group; G1 = 43 (100% schizophrenia)	36.3 (8.42)	25 (58.1%)	Reasons for Smoking Questionnaire; Reasons for Quitting scale; Readiness and Motivation to Quit Smoking Questionnaire	Reason for smoking: Addiction: 52.5%; Reasons for quitting, from highest to lowest: Health concerns; Desire for self-control; Perceived immediate reinforcement; Social influence; Received physician advice to quit: 81.4%.	82
Filia; 2014; Australia	To examine gender differences in perceived risks and benefits of quitting smoking in people diagnosed with psychosis presenting for a smoking cessation intervention study and compare risks and benefits with smokers in the general population.	200; 5 groups; G1 = 79 females (43% schizophrenia, 33% bipolar disorder, 24% other psychotic disorder), G2 = 121 males (67% schizophrenia, 17% bipolar disorder, 17% other psychotic disorder), G3 = 273 females (100% smokers in general population seeking cessation treatment), G4 = 300 males (100% smokers in general population seeking cessation treatment), G5 = 188 (non-treatment seeking smokers in general population)	G1 = 42.67 (9.93), G2 = 40.53 (11.76), G3-G5 = NA	G1 = 0 (0%), G2 = 121 (100%), G3 = 0 (0%), G4 = 300 (100%), G5 = NA	Perceived Risks and Benefits Questionnaire	Total risks: G1 < G3, G2 < G4, G1/G2 = G5; Weight gain: G1 < G3, G2 < G4, G1/G2 = G5; Increased negative affect: G1 = G3, G2 < G4, G1/G2 > G5; Poorer attention or concentration: G1 < G3, G2 < G4, G1/G2 = G5; Social ostracism: G1 < G3, G2 < G4, G1/G2 = G5; Loss of enjoyment: G1 < G3, G2 < G4, G1/G2 < G5; Increased cravings: G1 < G3, G2 < G4, G1/G2 = G5; Total benefits: G1 < G3, G2 < G4, G1/G2 = G5; Improved health: G1 < G3, G2 < G4, G1/G2 = G5; Improved wellbeing: G1 < G3, G2 < G4, G1/G2 > G5; Improved self-esteem: G1 < G3, G2 < G4, G1/G2 > G5; Improved finances: G1 < G3, G2 < G4, G1/G2 = G5; Greater physical appeal: G1 < G3, G2 < G4, G1/G2 > G5; Greater social approval G1 < G3, G2 < G4, G1/G2 = G5.	88
Forchuk; 2002; Canada	To determine whether individuals with schizophrenia were motivated to smoke to relieve psychiatric symptoms and relieve medication side-effects.	100; 1 group; G1 = 100 (100% schizophrenia)	36.2 (10.90)	72 (72%)	Modified Smoking Motives Questionnaire; Written responses to qualitative questions	Strongest motivators to smoke, from highest to lowest: Sedative effect; Control negative symptoms; Addiction; Control side effects of medication.	82
Himelhoch; 2009; USA	To determine whether individuals with schizophrenia and type 2 diabetes who smoke received appropriate care related to managing modifiable risk-factors associated with heart disease.	199; 2 groups; G1 = 61 (100% smokers with schizophrenia and diabetes), G2 = 34 (100% smokers with no serious mental illness and diabetes)	G1 = 48.6 (8.7), G2 = 49.9 (8.4)	G1 = 33 (54.1%), G2 = 18 (52.9%)	Reported receipt of smoking cessation counseling	Received physician advice to quit: G1 = G2	79
Hippisley-Cox; 2007; England	To determine whether coronary heart disease patients with schizophrenia were less likely than patients without mental illness to receive good quality care in accordance with UK agreed national standards.	127,932; 2 groups; G1 = 332 (100% schizophrenia), G2 = 127,231 (100% without mental illness)	Modal age groups = G1 = 65–74 years (30.7%), G2 = 75 years+ (44.9%)	G1 = 175 (52.7%), G2 = 75,283 (59.2%)	Clinician reported receipt of smoking cessation advice in past 15 months in smokers	Received physician advice to quit: G1 = G2	83
Kelly; 2012: USA	To compare knowledge and perception of smoking risks and motivation for quitting in smokers with and without schizophrenia	200; 2 groups; G1 = 100 (100% schizophrenia), G2 = 100 (no mental disorder)	G1 = 43.3 (11.4), G2 = 37.1 (10.6)	G1 = 71 (71%), G2 = 65 (65%)	Smoking Consequences Questionnaire; Reasons for Quitting Scale; Stages of Change Questionnaire	Reasons for quitting: Desire for self-control G1 = G2; Desire for immediate reinforcement: G1 < G2; Social pressure G1 > G2; Health concerns G1 < G2: Perceived smoking consequences: Stimulation: G1 > G2; Health risks: G1 < G2; Social facilitation: G1 > G2; Reduce negative affect: G1 = G2; Taste manipulation: G1 = G2; Appetite/weight control: G1 = G2; Craving/addiction: G1 = G2; Negative physical feelings: G1 = G2; Reduce boredom: G1 = G2; Social impression: G1 = G2.	83
Kourakos; 2014; Greece	To examine mental health patients' attitudes regarding smoking habits in the inpatient setting.	80; 1 group; G1 = 80 (65% schizophrenia/ schizoaffective disorders, 8% bipolar disorder, 28% other psychiatric illnesses)	52.55 (12.91)	54 (68%)	Patients' self-reported attitudes toward smoking	Received physician advice to quit: 25%; Staff should be able to smoke on the ward: 62.5% agreed; Visitors should be able to smoke with patients: 62.5% agreed; Seeing other patients smoke makes it difficult to quit: 62.5% agreed; Staff should encourage smokers to quit: 65% agreed.	50
Krishnadas; 2012; Scotland	To examine clinical variables associated with schizophrenia in an epidemiologically defined geographical area.	131; 2 groups; G1 = 70 (100% smokers with schizophrenia), G2 = 61 (100% non-smokers with schizophrenia)	G1 = 49.61 (14.48), G2 = 57.79 (17.21)	G1 = 47 (67.1%), G2 = 25 (41%)	Semi-structured questionnaire about smoking, smoking benefits and intentions of quitting	Reasons for smoking: Stress reduction: 60%; Manage depression or anxiety: 31%; Relieve loneliness: 16%; Socialize better: 14%.	83
Mann-Wrobel; 2011; USA	To understand the relationship between smoking and quit history, negative consequences due to smoking, stage of change, smoking temptation, and self-efficacy in people with schizophrenia participating in a smoking cessation trial.	41; 1 group; G1 = 41 (100% schizophrenia)	49.22 (8.0)	34 (82.9%)	Smoking Consequences Questionnaire; University of Rhode Island Change Assessment-Maryland	Perceived negative consequences of smoking, from highest to lowest: Health risks; Craving/addiction; Negative social impression. Perceived positive expectancies of smoking, from highest to lowest: Boredom reduction; Negative affect reduction; Social facilitation.	73
Spring; 2003; USA	To test two hypotheses: that patients with schizophrenia find smoking more rewarding then patients with depression; and, that patients with schizophrenia and patients with depression find smoking more rewarding than smokers without a psychiatric disorder other than nicotine dependence.	78; 3 groups; G1 = 26 (100% schizophrenia), G2 = 26 (100% depressive disorder), G3 = 26 (100% no psychiatric disorder)	G1 = 40.00 (10.85), G2 = 35.31 (11.13), G3 = 26.20 (11.69)	G1 = 19 (73%), G2 = 13 (50%), G3 = 21 (81%)	Decisional Balance Scale; Self-developed tool of preferences for engaging in smoking vs. other rewarding activities and magnitude of reward felt necessary for quitting	Decisional balance (pros minus cons): G1 = G2, G1/G2 > G3; Pros of smoking: G1 = G2, G1/G2 > G3; Cons of smoking G1 = G2 = G3; Alternative rewards were less favorable than smoking: G1 = G2, G1/G2 > G3; More rewards required to quit: G1 = G2, G1/G2 > G3	79
Tanriover; 2013; Turkey	To examine the frequency of smoking, smoking status, and smoking dependence in inpatients with schizophrenia, bipolar disorder, and major depression disorder.	160; 2 groups; G1 = 80 (53% schizophrenia, 31% bipolar disorder, 16% depressive disorder), G2 = 80 (100% no psychiatric diagnosis)	G1 = 36.83 (12.18), G2 = 37.65 (12.26)	G1 = 44 (55%), G2 = 39 (49%)	Self-developed tool to measure reason for tobacco use	Habit: G1 = 15%, G2 = 7.5% Addiction: G1 = 0%, G2 = 2.5%; Don't know: G1 = 1.3%, G2 = 1.3%; Society: G1 = 0%, G2 = 5%; Need: G1 = 1.3%, G2 = 0%; Desire to experiment: G1 = 1.3%, G2 = 17.5%; Boredom: G1 = 31.3%, G2 = 17.5%; Loneliness: G1 = 8.8%, G2 = 0%; Pleasure: G1 = 11.3%, G2 = 3.8%	63
Tidey; 2009; USA	To compare expected positive and negative smoking outcomes in smokers with schizophrenia, schizoaffective disorder, and equally-nicotine dependent smokers without psychiatric disorder, and to examine relationships between expected outcomes and intentions to quit smoking.	152; 3 groups; G1 = 46 (100% schizophrenia), G2 = 35 (100% schizoaffective disorder), G3 = 71 (no psychiatric disorder)	G1 = 45.1 (7.7), G2 = 43.9 (8.5), G3 = 44.5 (12.2)	G1 = 35 (76%), G2 = 19 (54%), G3 = 39 (55%)	Smoking Effects Questionnaire	Positive social effects: G1 = G2, G1 = G3, G2 > G3, G2 participants who did not intend to quit in 6 months > G2 participants who intended to quit in 6 months; Negative psychosocial effects: G1 = G2, G1 = G3, G2 > G3; Negative physical effects: G1 < G2, G1 = G3, G2 > G3	83
Tidey.; 2014; USA	To compare craving and withdrawal symptoms in smokers with schizophrenia and without schizophrenia across a 72-h period of abstinence, compare reinforcing effects of nicotine before and after abstinence, compare latency to smoking lapse, and examine predictors of lapse	55; 2 groups; G1 = 28 (100% schizophrenia, G2 = 27 (100% no psychiatric disorder)	G1 = 44.0 (10.6), G2 = 43.9 (10.8)	G1 = 16 (57%), G2 = 17 (63%)	Questionnaire on Smoking Urges—Brief form; Minnesota Nicotine Withdrawal Scale; Hedonic Rating Scale	Anticipated relief of negative affect: G1 > G2; Change in desire to smoke and withdrawal symptoms over 72 h abstinence: G1 = G2	83
Tulloch; 2016; Canada	To better understand the quit experience of smokers with and without psychiatric illness.	732; 2 groups; G1 = 302 (100% no psychiatric disorder), G2 = 18 (100% psychotic disorders)	G1 = 48.54 (11.01), G2 = 48.61 (10.83)	G1 = 188 (62.3%), G2 = 15 (83.3%)	List of reasons for relapsing smoking and motives and concerns about quitting	Cravings a concern for quitting: G1 < G2 (aOR = 4.16, 95%CI = 1.44, 12.06); Boredom a concern for quitting: G1 < G2 (aOR = 8.03, 95% CI = 2.25, 28.69)	67

*ANOVA, analysis of variance; aOR, adjusted odds ratio; CI, confidence intervals; G, group; N, number; NA, Not available; NRT, nicotine replacement therapy; SD, standard deviation; USA, United States of America*.

**Table 2:** Qualitative study characteristics and key findings.

**First author; year of publication; country**	**Study aims**	**Total sample size; N of relevant groups; sample size and group composition**	**Age in years (SD)**	**N male (%)**	**Assessment of barrier**	**Key findings**	**QualSyst score (%)**
Esterberg; 2005; USA	To examine the role of decisional balance in smoking and smoking cessation and the impact of external factors on smoking cessation attempts in people with schizophrenia, and differences between first-episode psychosis and chronic schizophrenia in relation to the transtheoretical model of change.	12; 1 group; G1 = 12 (100% schizophrenia)	Median = 25.5, Range = 19–43	10 (83%)	Semi-structured interview of pros and cons of smoking, beliefs about smoking cessation, external influences on smoking and quitting, and negative attitudes toward NRT	People living with schizophrenia smoke to relax and relieve negative symptoms Half the sample felt quitting was easy, with the other half citing quitting as challenging. Majority of participants believed there were more pros to smoking than cons. Lack of smoking cessation programs in hospitals, friend and family support to smoke, and negative views of NRT were additional barriers.	85
Goldberg; 1996; Canada	To obtain an overview of the smoking habits of people with schizophrenia, their stage of change, and their perceptions of influencing factors for smoking	105; 1 group; G1 = 105 (100% schizophrenia)	35, range 20–58	71 (68%)	Semi-structured interview on smoking habits, stage of change, and perceptions of factors that influence smoking behaviors	Most common barriers to quitting were addiction (53%), pleasure (20%), coping with symptoms/ clearing thought/ calming and relaxing effects (20%) and habit (19%). Most common motivators to quit were health concerns (33%), social support to address smoking (22%), cost (19%) and meaningful activities (16%).	45
Lawn; 2002; Australia	To describe smoking behavior experiences of people receiving mental health services, the relationship between smoking behavior and course of mental illness and management and quit attempts.	24; 1 group; G1 = 6 (100% schizophrenia)	G1–4 = 43	12 (50%)	Semi-structured interview exploring reasons underlying smoking behaviors	Main themes were the belief that smoking prevents relapse; provides control and freedom in otherwise powerless situation; health concerns not considered significant; alleviates positive symptoms; improves cognitive capacity and motivation; provides an identity; that peer and family encourages or accepts smoking; and that they would prefer to cut down than quit.	85

*G, group; N, number; NRT, nicotine replacement therapy; SD, standard deviation; USA, United States of America*.

### Cravings and addiction

The most commonly cited barrier to smoking cessation in people living with schizophrenia was cravings and addiction (nine studies). Two quantitative studies examining concerns associated with quitting found that cravings and addiction were the highest reported risks associated with smoking cessation (35, 36). Cravings were also frequently reported as a concern related to smoking cessation in two studies (37, 38), however one study indicated that cravings were not perceived as a reason for people living with schizophrenia to smoke cigarettes (39). Perceived or actual cravings associated with smoking abstinence were significantly higher among people living with schizophrenia compared with people without mental illness in three studies (36, 40, 41), yet were similar across groups in two studies (42, 43). One of the two studies in which cravings were reportedly higher in people living with schizophrenia than people without mental illness found that cravings increased over 72 h of abstinence in people living with and without schizophrenia, with no difference in the rate of increase across groups (41). Three studies examining whether sex and age was associated with cravings found no association (40, 42, 43). Two of the three studies which found higher perceived risk of cravings in people living with schizophrenia were based on small group sizes of 18 and 28.

### To reduce negative affect

A perceived increased risk of negative affect associated with quitting smoking was examined in seven studies. Perceived risk of increased negative affect was higher in people living with schizophrenia compared with people without mental illness in two studies (41, 43) and equal in three studies (36, 42, 44). One study reported that 31% of people living with schizophrenia smoked to reduce symptoms of anxiety and depression (45), while another reported that reducing negative affect would be the strongest motivator to smoke during abstinence (38). Filia et al. (43) found that females reported significantly higher perceived risk of negative affect associated with quitting compared with males.

### Social facilitation

Seven studies examined the relationship between smoking cessation and social facilitation in people living with schizophrenia. One study found that people living with schizophrenia were more likely to smoke to improve social functioning than people without mental illness (42). Another study found that people with schizoaffective disorder reported that positive social effects were related to lower intention to quit; a relationship that was not identified in people living with schizophrenia or without mental illness (44). One study reported that people living with schizophrenia felt equally as likely as people without schizophrenia to be socially ostracized if they were to quit smoking (43). Non-comparison studies also indicated that smoking was an important factor contributing to social comfort. One study found social factors were considered to be the second strongest factor contributing to smoking temptations in people living with schizophrenia (38). Krishnadas et al. (45) reported that 14% of respondents reported smoking to socialize better, while Kourakos and Koukia (46) found that 83% of psychiatric inpatients with schizophrenia believed that visitors should be allowed to smoke with patients. Neither age (42) nor did sex (43) appear to be associated with perceived social facilitation as a motivator to smoke.

### Stress management

Five studies reported on the impact of smoking on stress reduction in relation to smoking cessation. One study reported that people living with schizophrenia were significantly more likely to smoke to reduce stress compared with people from the general population (40). A second study found no differences in ratings of stress as a concern related to quitting smoking between people living with schizophrenia and people without mental illness (36). However, the small group size of people living with schizophrenia may have reduced the likelihood of identifying true group differences (i.e., Type 2 error). Two other studies identified stress reduction as the main reason for smoking (35, 37), while another found that 60% of people living with schizophrenia smoke to relax (45). Two studies found that sex and age were not associated with stress management as a motivator to smoke (40, 42).

### Concern for health risks

We identified eight studies examining perceptions of health risks as facilitators to smoking cessation in people living with schizophrenia. In four of these studies, people living with schizophrenia reported perceptions that the health benefits associated with quitting smoking were equal to perceptions of people without mental illness (36, 40, 43, 44). One included study reported that concern for health was the highest rated reason to quit smoking (35) and another study reported that people living with schizophrenia perceived smoking-related health implications were the most negative consequences of smoking (38). However, two studies found that people living with schizophrenia reported lower health-related concerns than people without mental illness (42, 47). A greater proportion of people with recent onset psychosis compared with smokers without mental illness believed smoking had fewer adverse health effects, including for stroke, brain damage, lung disease, heart disease, cancer, and miscarriage (47). No differences in concern for health between males and females or relating to age were identified (40, 42, 43).

One study compared picture and video health warnings on perceived effectiveness and emotional valence and arousal in people living with schizophrenia and people without mental illness (47). People living with schizophrenia perceived the health warnings as more effective than controls. Perceived effectiveness of health warnings was positively associated with the emotional valence and arousal reported by participants.

### Physician advice to quit smoking

Seven studies reported on advice to quit by a health professional. One study reported that people living with schizophrenia were less likely to have received instruction for smoking cessation than people without mental illness (48), two found equal levels of advice provided to people living with and without schizophrenia (49, 50), while a fourth study found mixed results depending on the type of advice provided (51). The median percentage of people living with schizophrenia reporting receipt of advice to quit by a health professional was 80%, with a range between 25 and 94% (35, 46, 49–51), while the median for people without schizophrenia was 83%, ranging from 79 to 98% (49–51). Four studies comparing rates of physician advice were conducted in samples with smoking-related comorbidities, such as cardiovascular heart disease or diabetes, or in unique populations, such as members of Veterans' Affairs.

Brown et al. (52) examined the perceptions of mental health clinicians in providing the “5 A's” (ask, advise, assess, assist, arrange) of smoking cessation advice to their patients, which included a majority percentage of people living with schizophrenia. Clinicians rated their perceived lack of interest among patients of all diagnoses to discuss smoking and/or smoking cessation, too many demands on staff already to begin a new practice, too time demanding to carry out 5 A's, and staff skepticisms about the value of the 5 A's as the strongest barriers preventing implementation of the 5 A's.

### Systemic barriers

Patients with schizophrenia in a psychiatric inpatient unit with few rules regarding smoking on the ward were generally supportive of the hospital's smoking policy (46). Seventy-five per cent agreed that the liberal ward rules about smoking were correct, while 63% believed that visitors and/or staff should be allowed to smoke on the ward. However, 90% of participants reported that it was too difficult to quit, with 63% believing that seeing other patients smoke would make it difficult to quit and 65% reporting that staff should set a good example.

### Social pressure to quit

Two studies identified social pressures to quit smoking as reportedly higher among people living with schizophrenia compared with people without mental illness (40, 42), however another two studies found equal levels of social pressure across groups (36, 43). No study identified a link between age or sex and social pressure to quit among people living with schizophrenia (40, 42). One study also reported that people living with schizophrenia who perceived their friends would be more likely to approve of NRTs or smoking cessation medication had higher intentions to quit smoking using these two pharmacotherapies (53).

### Additional barriers and facilitators to smoking cessation

Reduction of boredom was another common reason people living with schizophrenia smoke. Five studies examined boredom as a reason to smoke, with one study finding people living with schizophrenia were eight times more likely than people without mental illness to report boredom as the reason for smoking (36). Three studies reported that boredom was the highest rated reason that people living with schizophrenia smoked cigarettes (38, 39, 42). Yet one of these studies found no difference between people living with schizophrenia and people without mental illness in ratings of boredom as a reason to smoke cigarettes (42).

Five studies examined stimulation as a reason to smoke, with two studies identifying people living with schizophrenia as significantly more likely to smoke for stimulation or arousal when compared with people without mental illness (40, 42). Two studies found no difference between people living with and without schizophrenia (43, 44). Stimulation as a reason to smoke was not associated with age or sex (40, 42).

People living with schizophrenia were equally as likely as people without mental illness to smoke for the purpose of managing weight in four studies (36, 42–44). Mann-Wrobel et al. (38) reported prevention of weight gain as the least important positive consequence of smoking among people living with schizophrenia. One study found that females were more likely than men to report higher perceived risk of weight gain associated with quitting (43).

One study compared perceptions of the pros and cons of smoking in three groups comprised separately of people living with schizophrenia, depression, or without mental illness (54). People living with schizophrenia and depression reported similar levels of pros and cons of smoking, with the combined schizophrenia and depression group reporting significantly more pros of smoking than the control group and an equal number of cons. The combined schizophrenia and depression group also reported that smoking was more rewarding than a higher number of alternative pleasurable activities than the control group. People living with schizophrenia reported requiring more rewards, such as coffee or money, to quit smoking than people without a mental illness. Age was not associated with perceived pros and cons of smoking.

### Qualitative findings

The qualitative studies supported the quantitative findings. Esterberg and Perneger (55) reported that people living with schizophrenia smoke to relax, gain relief from negative symptoms, and relieve boredom. Similar to Spring et al. (54), Esterberg and Perneger (55) reported that the majority of participants believed there were more pros to smoking than cons. Lawn et al. (56) found that people living with schizophrenia expressed little concern for their physical health, preferring to smoke as a way to manage positive symptoms and improve problem solving skills. In contrast, Esterberg and Perneger (55) reported that people living with schizophrenia were aware of the negative health implications, including cancer, and reduced engagement in physical activity, and the financial burden of smoking. Health concerns and a desire to increase self-esteem prompted eleven of the twelve participants to attempt quitting, yet feelings of tension and nervousness led them to begin smoking again. Two studies reported that people living with schizophrenia felt that their family, friends, and health professionals provided little reinforcement for them to quit (55, 56).

Following cravings as the most commonly reported barrier to smoking cessation (53%), Goldberg et al. (57) reported that pleasure and enjoyment associated with smoking, as well as coping with symptoms of anxiety were both reported as barriers to quitting by 20% of people living with schizophrenia. Habit (19%), boredom (17%), and a social environment associated with pressure to smoke and that provided little support to quit (13%) were other important barriers to smoking cessation. Other barriers reported in the qualitative studies were that people living with schizophrenia smoke for a sense of identity as it has shaped their development and contributes to their current sense of self and to feel freedom from their powerlessness in deciding their future (56). People living with schizophrenia also reported that the lack of smoking cessation programs in hospitals was also a barrier to quitting (55). People living with schizophrenia generally viewed NRT as negative and associated NRT with a sense of increasing rather than decreasing cravings, being unhealthy, unwanted side-effects and viewed NRT as unnecessary to quit (55). Participants believed that reducing, rather than quitting, would be a more realistic goal (56).

## Discussion

We systematically reviewed barriers to smoking cessation in 23 studies including 3,257 people living with schizophrenia. People living with schizophrenia reported that the main reasons for smoking are to manage cravings and addiction, as well as negative symptoms such as their desire to reduce negative affect, facilitate social relationships, manage stress, and relieve boredom. Medical professionals may be less likely to provide smoking cessation advice to people living with schizophrenia when compared with people without mental illness. The social networks of people living with schizophrenia may show little support for smoking cessation, which may reduce the likelihood people living with schizophrenia will attempt to quit smoking. While people living with schizophrenia appeared to be as aware of the smoking-related health risks as people without mental illness, they may be less likely to act on this awareness. Overall, it appears that people living with schizophrenia experience a greater number of barriers to smoking cessation than people without mental illness.

Cravings and addiction to smoking were reported as one of the main reasons for smoking in people living with schizophrenia in five studies (35–38, 57). The highly addictive properties of nicotine may have a greater influence on people living with schizophrenia compared with those without mental illness (36, 40, 41). Past research suggests that people living with schizophrenia have a higher nicotine dependence than people in the general population (1). These findings indicate the need to address the physical addiction to smoking with NRT, such as patches or gum, or smoking cessation medications, such as varenicline or bupropion.

While pharmacotherapy may increase smoking cessation rates, one qualitative study included in this review highlighted the strong negative attitudes that people living with schizophrenia have toward NRT, such as the belief that NRT increased cravings rather than decreased them, that NRT made them feel sick and that NRT was unhealthy (55). Negative attitudes toward NRT are also common among smokers without schizophrenia (58, 59). These findings are concerning, as we identified one study examining people living with schizophrenia which found that positive attitudes toward NRT held the strongest relationship with greater intention to use NRT (53). Attitudes toward pharmacotherapy may be an important psychosocial component of combined pharmacological and psychosocial interventions. Further intervention research examining the role of psychosocial support in promoting adherence to pharmacological treatments, including NRT, in people living with schizophrenia is required.

People living with schizophrenia also reported smoking to reduce negative affect, relieve boredom, manage stress, and facilitate social relationships. These reasons for smoking all target negative symptoms of schizophrenia, supporting previous research that has identified a link between greater negative symptom severity and increased smoking rates or nicotine dependence (2, 60, 61). However, in contrast to the views of people living with schizophrenia, smoking cessation is associated with lower levels of stress, anxiety, and depression than continued smoking (62). Psychoeducation on the effects of smoking on negative symptoms and other psychological treatment to manage negative symptoms may help reduce smoking rates in people living with schizophrenia. The National Institute for Health Care and Excellence (NICE) guidelines recommend CBT as a psychological treatment for schizophrenia (63). A 2014 meta-analysis found that psychological treatments, such as CBT, and pharmacological treatments, such as antidepressants and second-generation antipsychotics, significantly reduced negative symptoms in people living with schizophrenia (64).

Only one study has examined CBT to promote smoking cessation in people living with schizophrenia, which found significantly higher abstinence and reduction rates at 3, 6, and 12 months in participants who attended all 8 treatment sessions when compared with participants receiving treatment as usual (8). Continued research examining CBT on smoking cessation in people living with schizophrenia is required. Behavioral activation, an important component of CBT, is an effective standalone treatment for depression and may have a unique influence on negative symptoms of schizophrenia (65, 66), however no research examining the effect of behavioral activation on smoking cessation has been conducted in people living with schizophrenia. Future research examining the mediating role of negative symptoms in treatment effect on smoking cessation will enhance the evidence linking smoking with negative symptoms.

Most people living with schizophrenia appear to have some awareness of the health risks associated with smoking (36, 40, 43, 44) and health risks are often cited as a facilitator to quit smoking (35, 38). Health concerns are also considered the most important reason to quit smoking among the general population (25, 67). Health professionals should continue to raise awareness of the health risks of smoking among people living with schizophrenia. A review found that half of the reviewed interventions to improve health literacy among primary care patients led to reduced smoking rates, with significant outcomes most commonly associated with information that was provided via individual counseling or through written resources (68).

Considering our findings that people living with schizophrenia rate health risks as an important reasons to quit smoking, the authors strongly encourage health professionals to follow the 5 A's (ask, assess, advice, assist, arrange) when working with people living with schizophrenia. Unfortunately, we found mixed evidence from the seven studies examining receipt of advice to quit from a physician. Two studies found that people living with schizophrenia were less likely to be advised to quit smoking compared with people without mental illness (48, 51), while a third non-comparison study found that only 25% of people living with schizophrenia were advised to quit (46). Barriers impeding clinicians' likelihood of administering smoking cessation advice to people with mental illness included a perceived lack of interest to quit in patients and insufficient staff and time to provide such support (52). Training that aims to improve physicians' attitudes and perceived competence to deliver the 5 A's may increase their likelihood of delivering smoking cessation advice, especially when training is combined with other interventions components, such as patient counseling, patient access to tailored information resources, and free NRT (69).

### Strengths and limitations

Our review amalgamated a diverse range of studies that included individuals from a variety of geographical locations and community or clinical settings, thereby enhancing the generalizability of the findings. However, we failed to identify eligible articles from Asia, South America, or Africa. Thus, our findings may be limited to people living with schizophrenia residing in Western societies.

We limited our review to include samples in which people living with schizophrenia were the majority. Six studies included a mixed sample of people living with schizophrenia or other mental illnesses, yet 91.6% of participants in groups including people living with schizophrenia were diagnosed with schizophrenia. Thus, we can be confident that our findings are specific to the target population and that they overcome limitations of previous reviews that combine results for people living with severe mental illness, such as bipolar disorder and severe depression.

We chose to include articles that contained baseline data from intervention studies. In doing so, we increased the evidence base and quality of available evidence. Yet, we may have biased the sample by including studies that excluded people living with schizophrenia who were ineligible to participate in the intervention. Common exclusion criteria of intervention studies included in our review were cognitive impairments, diagnosis of a medical condition precluding use of NRT, or brain injury. Our findings may not extend to people living with schizophrenia with these comorbidities.

The 23 articles included in our study held a number of limitations that may also affect the reliability of our findings. Nine studies (39%) included samples with fewer than 50 participants and eight studies (35%) had sample sizes between 51 and 100. Small sample sizes reduce the reliability and generalizability of the findings. Six of the 20 (30%) quantitative studies did not include a control sample limiting our understanding of whether the findings were different for people without schizophrenia or other mental illnesses. We found that there was inconsistent use of validated assessment tools. Three studies used the Reasons For Quitting scale (70), two used the Reasons for Smoking Questionnaire (71) and two used the Smoking Consequences Questionnaire (72). Most other studies used assessment tools purposively developed for their study. The reliability of evidence will be improved with more studies using the same assessment tool. Future research may also wish to validate these assessment tools in people living with schizophrenia and should continue to explore reasons for smoking and quitting that are not addressed in these assessment tools.

### Recommendations for future research and practice

Our review highlighted the presence of a number of important psychosocial barriers and facilitators to smoking cessation in people living with schizophrenia. A 2013 Cochrane review found only five randomized controlled trials examining psychosocial intervention effects on smoking cessation in people living with schizophrenia (7). Our findings can be used to inform the development of psychosocial interventions that are combined with pharmacological treatments. Future research that examines the effects of separate treatment components (e.g., cognitive vs. behavioral components of CBT) and the role of possible mediators, such as reduced negative symptoms or baseline nicotine dependence, will continue to inform the literature of the mechanisms facilitating smoking cessation among people living with schizophrenia.

Our findings strongly support current smoking cessation guidelines for physicians to advise people living with schizophrenia that cravings, withdrawal symptoms, nicotine dependence, and health risks will continue if they continue to smoke (73). Physicians may also assist people living with schizophrenia quit smoking by providing information on smoking-related health risks, delivering brief counseling or MI, or referring patients to specialized smoking cessation services. Future research may be required to examine strategies to overcome barriers reducing physicians' likelihood of providing advice and assistance, such as through brief professional training programs. In addition to increasing physicians' likelihood of providing advice and assistance, future research should assess the impact of information resources educating people living with schizophrenia of the smoking-related health risks on their motivation to quit smoking and smoking cessation rates.

While the evidence is far from conclusive, electronic nicotine devices (i.e., e-cigarettes) may help reduce smoking rates by managing both the physical and psychological addictive properties of cigarettes (74). The regulation of e-cigarettes and nicotine liquids for use in e-cigarettes varies worldwide. In some countries, nicotine liquids cannot be purchased without a prescription. Ongoing research in this area is needed to help determine whether e-cigarettes increase smoking cessation rates, whether people living with schizophrenia are open to using e-cigarettes, and how physicians may influence the uptake of e-cigarettes for smoking cessation purposes.

## Conclusion

Our systematic review found a range of important barriers and facilitators to smoking cessation in people living with schizophrenia. Addiction and cravings appear to be a primary reason why people living schizophrenia smoke cigarettes, yet there is also strong evidence that they smoke to manage features of negative symptoms, such as stress, negative affect, boredom, and social isolation. Health professionals also play an important role in smoking cessation for people living with schizophrenia and should support quitting in people living with schizophrenia as much as they do for people without mental illness. The barriers and facilitators identified in this review should be used to inform the development of targeted psychosocial components of smoking cessation interventions for people living with schizophrenia.

## Author contributions

AL, ES, OW, and BB contributed to the study conception and design. AL, ES, and OW collected, analyzed, and interpreted data. AL wrote the first draft of the manuscript. AL, ES, OW, and BB contributed to critical revision of the manuscript, read and approved the submitted version.

### Conflict of interest statement

The authors declare that the research was conducted in the absence of any commercial or financial relationships that could be construed as a potential conflict of interest.
